# Evaluating Telepsychiatric Assessment Satisfaction in Children and Adolescents With Autism Spectrum Disorder and Attention-Deficit/Hyperactivity Disorder and Their Caregivers: Randomized Controlled Trial

**DOI:** 10.2196/69791

**Published:** 2025-09-05

**Authors:** Shunya Kurokawa, Yuko Kawade, Kensuke Nomura, Nana Hosogane, Takashi Nagasawa, Yu Matsumoto, Shuichi Morinaga, Yuriko Kaise, Ayana Higuchi, Akiko Goto, Naoko Inada, Masaki Kodaira, Taishiro Kishimoto

**Affiliations:** 1 Center for the Promotion of Interdisciplinary Research in Medicine and Life Science, Keio University School of Medicine, Mori JP Tower F7, 1-3-1, Azabudai, Minato-ku, Tokyo, 160-0041, Japan, 81 353633219; 2 Department of Child Psychiatry, Shimada Ryoiku Medical Center for Challenged Children, Tokyo, Japan; 3 Department of Child and Adolescent Mental Health, Aiiku Clinic, Tokyo, Japan; 4 Department of Child and Adolescent Psychiatry, Tokyo Metropolitan Children's Medical Center, Tokyo, Japan; 5 Department of Neuropsychiatry, Keio University School of Medicine, Tokyo, Japan; 6 Tsurugaoka Garden Hospital, Tokyo, Japan; 7 Department of Clinical Psychology, Taisho University, Tokyo, Japan

**Keywords:** attention-deficit/hyperactivity disorder, autism spectrum disorder, telepsychiatry, telehealth, caregivers, ADHD, ASD, mobile phone

## Abstract

**Background:**

Children and adolescents with attention-deficit/hyperactivity disorder (ADHD) and autism spectrum disorder (ASD) often face structural and psychological barriers in accessing medical care, including economic costs, long wait times, and stress of attending new medical environments. The COVID-19 pandemic accelerated the adoption of telehealth services to overcome these challenges. However, few studies have assessed the satisfaction levels of children and adolescents diagnosed with neurodevelopmental disorders and their caregivers when they use telepsychiatry, particularly in Japan.

**Objective:**

This study aimed to evaluate satisfaction by conducting telepsychiatric assessments in children and adolescents diagnosed with ADHD or ASD and their caregivers and to identify factors associated with higher satisfaction levels.

**Methods:**

A total of 68 patients aged 6‐17 years with a confirmed diagnosis of ADHD or ASD and their caregivers participated in this study. The participants were recruited from Keio University Hospital and four collaborating institutions in Japan. Each patient and their caregiver underwent two assessment sessions, one face-to-face and the other via telepsychiatric assessment (a remote video tool), in a randomized order. Upon completing both assessments, the participants completed a satisfaction questionnaire using a 5-point Likert scale that covered aspects such as audio and video quality, seamless communication, perceived warmth, reduced burden, and the ability to behave naturally. Spearman rank correlation coefficients and multiple regression analyses were performed to identify factors associated with overall satisfaction.

**Results:**

Among the patients, 70% (47/67) reported being “satisfied” or “very satisfied” with the telepsychiatric assessment, and 88% (60/68) of caregivers reported similar satisfaction levels. Multiple regression analysis showed that in children, high satisfaction was associated with seamless viewing of the screen, reduced burden of hospital visits, and the ability to speak naturally during the assessment. For caregivers, visual clarity and the child’s natural behavior were crucial factors.

**Conclusions:**

Telepsychiatric assessments are an effective and practical option to provide care for children and adolescents diagnosed with ADHD or ASD and their caregivers, offering high levels of satisfaction. Technical reliability and reduced travel burden significantly contributed to positive experiences. However, ensuring that children and adolescents behave naturally and feel a sense of warmth during remote assessment is crucial to maximizing their satisfaction. Telepsychiatric services can enhance the quality of health care, making them valuable supplementary tools for clinical practice.

## Introduction

Neurodevelopmental disorders, including attention-deficit/hyperactivity disorder (ADHD) and autism spectrum disorder (ASD), are complex disorders caused by genetic and environmental factors. Early detection and intervention are crucial to prevent comorbidities, such as anxiety and depression [1,2]. Children and adolescents with neurodevelopmental conditions and their families frequently encounter structural and psychological barriers to obtaining medical treatment.

Structural barriers include economic costs [3,4], taking time off work [5], lack of treatment availability owing to a shortage of trained psychiatrists [6,7], and long-distance travel to specialized clinics [8]. Psychological barriers that impede engagement with neurodevelopmental disorder services include direct caregiver distress, the stigma associated with ASD and ADHD and their treatment [9,10], and low self-efficacy in accessing and implementing treatment [11]. For children and adolescents with neurodevelopmental disorders, traveling to medical appointments and being in unfamiliar medical settings can worsen behavioral problems and increase psychological stress [12].

In recent years, the development of information and communication technology has led to the widespread adoption of telehealth, in which individuals communicate via video calls over long distances. During the COVID-19 pandemic, in-person medical care was limited owing to the increased risk of infection from face-to-face contact. As an alternative, telepsychiatry proved to be an effective method in psychiatric care. This is attributable to most psychiatric assessments and treatments relying on medical interviews, behavioral observations, and patient conversations.

Numerous studies have reported high satisfaction with the use of telepsychiatry in the field of psychiatry [13,14]. However, few studies have focused on children with neurodevelopmental disorders. The treatment setting for children with ASD conditions and intellectual developmental disorders at a pediatric hospital in the United States during the COVID-19 pandemic received a 91% satisfaction rate for telepsychiatric health assessments [8]. Reisinger et al [15] investigated the satisfaction of telehealth assessments in children with ASD. They found that 88% of caregivers reported being satisfied with the assessments, with some implications for factors related to satisfaction. Galvin et al [16] surveyed caregivers of children with ADHD in Ireland regarding their satisfaction with the remote consultations they had received. Most participants (62.3%) reported being comfortable communicating with their health care professionals via telehealth consultations, and only 34.4% reported that their child was comfortable communicating through their telehealth experience. However, responses were not directly obtained from the children.

Despite the above reports, few studies have investigated satisfaction with telepsychiatry among children with ASD and ADHD and their caregivers, particularly in Japan. Studies on the advantages and disadvantages of using remote assessment for neurodevelopmental disorders in children remain limited, particularly regarding the satisfaction of children undergoing the assessment. Previously, we reported the reliability of remote ADHD assessment compared to face-to-face assessment in children with ASD or ADHD and their caregivers [17]. We used intraclass correlation coefficients to confirm that the assessments conducted remotely were comparable to those conducted face-to-face. This study aimed to examine the acceptance of remote evaluations by end users, particularly children with ASD or ADHD and their caregivers, and identify the factors associated with higher satisfaction.

## Methods

### Participants

Kurokawa et al [17] provide a detailed description of the related research methodologies. Patients were recruited at the Keio University Hospital and four collaborating institutions (Shimada Ryoiku Medical Center for Challenged Children, Aiiku Clinic, Tokyo Metropolitan Children’s Medical Center, and Tsurugaoka Garden Hospital). The inclusion criteria were as follows: (1) confirmation of the Diagnostic and Statistical Manual of Mental Disorders diagnoses of ADHD and ASD, (2) age 6‐17 years at the time of obtaining consent, and (3) stable treatment for 3 months before obtaining consent. The exclusion criteria were as follows: (1) either the child or caregiver had a hearing or visual impairment that made it challenging to use remote tools, even with corrective devices, such as glasses or hearing aids; (2) caregivers did not have the information related to the participants’ early childhood; (3) comorbid symptoms, such as hallucinations and delusions, made it challenging to involve the participants in the study, as determined by the clinician; and (4) there were plans to initiate new treatments, such as pharmacotherapy or psychotherapy, during the observation period. This study was conducted in line with CONSORT (Consolidated Standards of Reporting Trials) guidelines (Checklist 1).

### Ethical Considerations

The study was conducted in accordance with the Declaration of Helsinki and the Ethical Guidelines for Medical and Health Research Involving Human Subjects (Japan). Informed consent was obtained from all the caregivers. For children, an informed consent form that was tailored to their age and understanding was used, and written confirmation of their consent was obtained after careful explanation. Regarding confidentiality, a designated data manager assigned each participant a unique study identification number and managed all records exclusively under this number thereafter. The correspondence table linking personal identifiers to study IDs was stored either in a locked cabinet within the Department of Psychiatry and Neurobiology at Keio University School of Medicine or on a password-protected computer with restricted access. All subsequently collected data and background patient information (eg, diagnosis, sex, and age) were handled solely by study ID and were completely separated from personal identifiers, such as participants’ names, dates of birth, patient numbers, or addresses. The study protocol was approved by the Ethics Committee of Keio University School of Medicine (20190301). This study is registered in the UMIN Clinical Trial Registry (UMIN000039860).

### Study Procedure

For baseline evaluation, the Autism-Spectrum Quotient–Japanese version for children, Conners 3 Japanese version to evaluate ADHD symptoms by caregivers, Strengths and Difficulties Questionnaire to assess social adaptation, and Short Sensory Profile to assess sensory characteristics were administered. Background information and data such as age, sex, diagnosis, medication, duration of illness, and intelligence test results were collected from medical records.

Patients and caregivers underwent assessments twice on different days, either face-to-face or remotely, in randomized order. The remote smartphone assessment tool “Curon” (MICIN Co Ltd) was used for the telepsychiatric assessment. The participants were seated in front of a smartphone in their homes and introduced to a remote evaluator. Remote evaluators administered their evaluations from a room at the Keio University Hospital using a personal computer. Assessments began after confirming the absence of interruptions in the video or audio environment. None of the raters was involved in the participants’ assessments or treatment during their usual visits.

Upon completing the face-to-face and remote assessments, the participants were provided with a questionnaire to report their satisfaction with the assessment.

### Satisfaction Questionnaire

The survey assessed the levels of satisfaction and feasibility associated with the use of telehealth tools during assessment interviews. A 5-point Likert scale ranging from 1=strongly disagree to 5=strongly agree was used to collect responses. The questions covered include: (1) audibility during telehealth assessments, (2) clarity of video presentation, (3) communication issues compared to in-person assessments, (4) lack of warmth or personal touch that made the patient feel uneasy, (5) perceived benefits of less time and burden compared to in-person hospital visits, and (6) whether the child could behave naturally compared to a face-to-face assessment. The participants were allowed to provide free-text comments on each item to illustrate their experiences.

### Statistics

Analyses were conducted to identify the crucial factors related to overall satisfaction with the telehealth tools used by patients and their caregivers. Spearman rank correlation coefficients were used to identify factors strongly associated with overall satisfaction with remote assessment among patients and caregivers. These factors included age, sex, diagnosis, severity of ASD and ADHD, other survey items, waiting time for the initial consultation, and travel time to clinic appointments. The Wilcoxon rank sum test was used for binary categorical data.

Subsequently, multiple regression analysis was conducted to examine the factors associated with overall satisfaction. Variables showing strong correlations in the preliminary exploratory data analyses and those based on theoretical grounds were selected using a forced-entry method to develop the models. Separate models were developed for the patients and caregivers. For patients, the analysis included age, sex, main diagnosis, clarity of auditory and visual reception, difficulty in expression, sense of warmth, perceived reduction in time and psychological burden compared with usual care, and seamless speaking naturally. The variance inflation factor was calculated to address multicollinearity, with variables exceeding a variance inflation factor of 10 being excluded from the model. Data analysis was performed using SPSS Statistics (version 28; IBM Corp). The significance level was set at .05. All statistical tests were 2-tailed.

## Results

**Figure 1.**
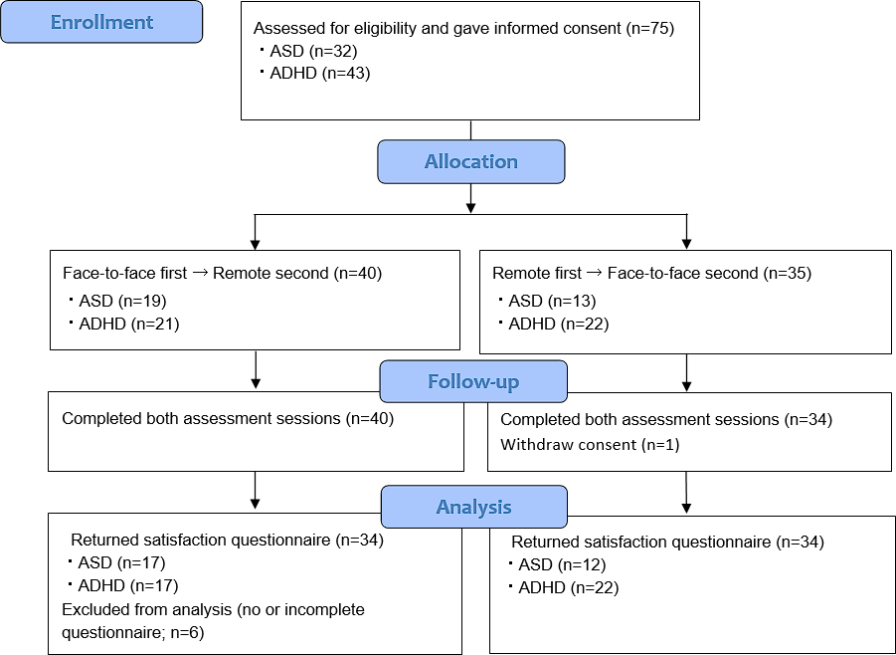
CONSORT diagram of participant flow through the study. ADHD: attention-deficit/hyperactivity disorder; ASD: autism spectrum disorder; CONSORT: Consolidated Standards of Reporting Trials.

The study initially enrolled 75 participants but concluded with 74, as one participant dropped out owing to scheduling conflicts over the 3-month period. Upon excluding 16 individuals for whom comprehensive satisfaction survey data were not obtained, 68 children and their caregivers were included in the analysis (Figure 1).

Table 1 presents the mean data. The sex distribution was predominantly male, with 53 (78%) male and 15 (22%) female participants. The primary clinical diagnoses were ASD in 29 (43%) individuals and ADHD in 39 (57%), with 18 (27%) participants having comorbid ASD and ADHD.

**Table 1.** Participant demographics.

	All (n=68)	ADHD^a^ (n=39)	ASD^b^ (n=29)	*P* value
Sex (female), n (%)	15 (22)	10 (26)	5 (17)	.41
Age (years), mean (SD)	10.2 (2.4)	10.5 (2.4)	9.8 (2.2)	.19
SDQ^c^, mean (SD)	21.52 (4.88)	20.55 (4.99)	22.68 (4.57)	.09
AQ^d^, mean (SD)	24.30 (7.32)	22.42 (6.47)	26.86 (7.75)	.02
Conners 3, mean (SD)	110.35 (37.05)	104.24 (67.60)	117.79 (35.64)	.15
SSP^e^, mean (SD)	70.65 (20.60)	66.84 (20.63)	75.52 (19.87)	.09
CARS-2^f^ (face-to-face), mean (SD)	36.15 (4.95)	34.36 (4.61)	38.50 (4.43)	<.001
ADHD-RS-IV^g^ total (face-to-face), mean (SD)	27.29 (10.02)	26.55 (10.57)	28.29 (9.33)	.48
ADHD-RS-IV hyperactivity/impulsiveness (face-to-face), mean (SD)	7.91 (6.20)	7.63 (6.68)	8.29 (5.58)	.67
ADHD-RS-IV total inattention (face-to-face), mean (SD)	19.38 (5.31)	18.92 (5.47)	20.00 (5.11)	.41

a ADHD: attention-deficit/hyperactivity disorder.

b ASD: autism spectrum disorder.

c SDQ: Strengths and Difficulties Questionnaire.

d AQ: Autism Spectrum Quotientt.

e SSP: short sensory profile.

f CARS-2: Childhood Autism Rating Scale-2.

g ADHD-RS-IV: attention-deficit/hyperactivity disorder rating scale-IV.

**Figure 2.**
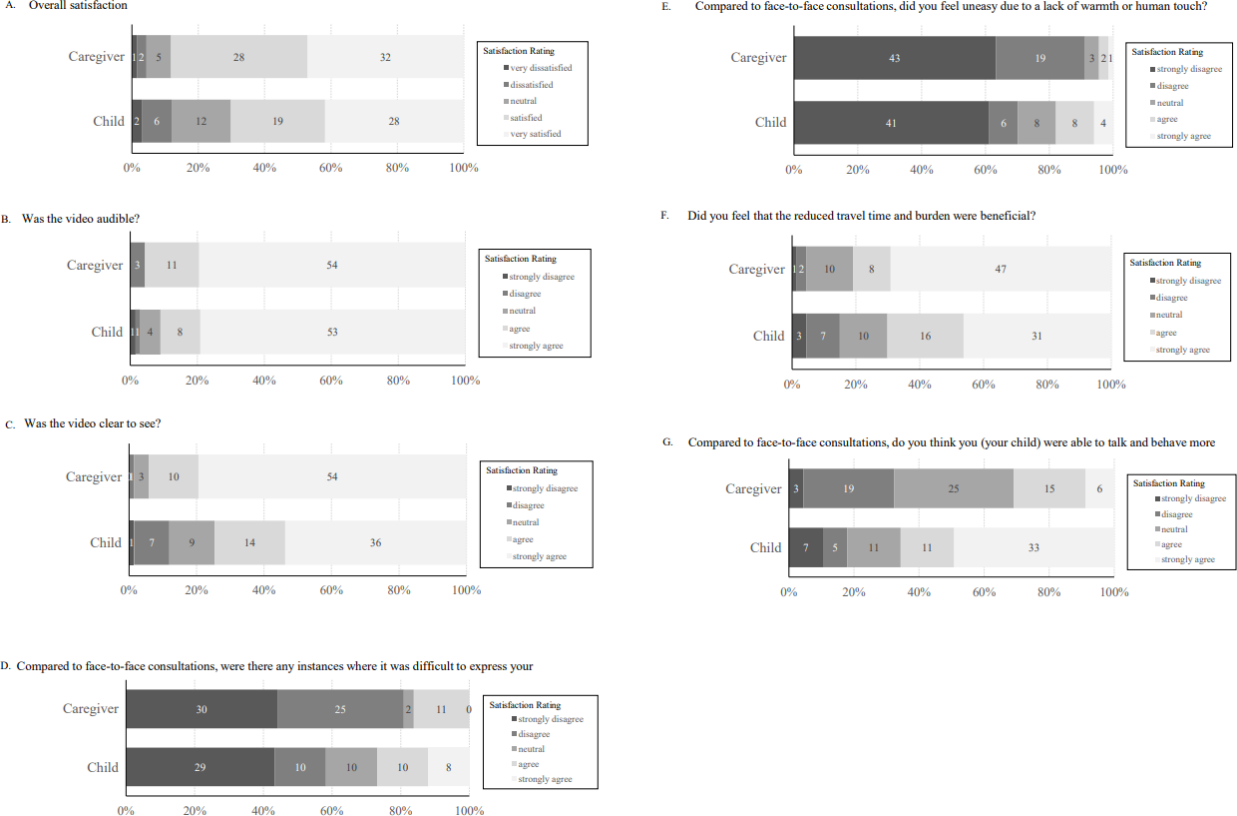
Responses of patients and their caregivers to each question. (A) Overall satisfaction, (B) seamless hearing (voice), (C) seamless seeing (video), (D) comparison of face-to-face consultation with seamless expression of message, (E) comparison of face-to-face consultation with warmth or physical touch, (F) comparison of face-to-face consultation with reduced time travel, and (G) comparison of face-to-face consultation with children’s ability to walk and behave naturally.

Figure 2 shows the responses of patients and their caregivers to each question. Additionally, 70% (47/67) of children reported being “satisfied” or “very satisfied.” Among caregivers, 88% (60/68) reported being “satisfied” or “very satisfied.” Regarding seamless hearing, 91% (61/67) of the children answered that they could “hear well,” “hear very well.” For caregivers, 96% (65/68) responded with “hear well” or “hear very well.” Regarding screen visibility, 75% (50/67) of children found it “easy to see” or “very easy to see.” Similarly, 94% (64/68) of caregivers found the screen “easy to see” or “very easy to see.” Regarding difficulties in communicating during the interviews, 58% (39/67) of children responded “not at all” or “no” regarding whether it was challenging to express themselves compared to face-to-face assessments. Among caregivers, 81% (55/68) responded “not at all” or “no.”

When asked if they felt uneasy owing to a lack of warmth or connection compared to face-to-face assessments, 70% (47/67) of children answered “not at all” or “no.” For caregivers, 91% (62/68) responded “not at all” or “no.”

Regarding the reduced time and burden of traveling for appointments, 70% (47/67) of children felt it was “beneficial” or “very beneficial.” Among caregivers, 81% (55/68) reported it was “beneficial” or “very beneficial.” Regarding their feeling that they could act naturally, 66% (44/67) of children responded “agree” or “strongly agree.” However, only 31% (21/68) of the caregivers responded “agree” or “strongly agree.” Regarding security concerns, 76% (52/68) of the caregivers responded “not at all” or “no.”

In the free text, responses highlighted that for some children and caregivers, being at home facilitated relaxation during the assessment interviews, indicating that the home environment positively affected the participants’ experience.

Many responses from the free text indicated that the caregivers were relieved about saving time. They reported that they had to consider the time for the patient’s appointment, as well as the travel time, while managing other children’s schedules. Some caregivers expressed psychological stress as follows: “I have to take my child out of school for appointments, and I always struggle with whether I must prioritize their medical care or their education because the visits are often on weekdays.”

The caregivers had different opinions, including how “the child’s behavior was too relaxed during the remote assessment and differed from their in-person assessment,” whereupon they questioned the validity of the assessment. The difficulty of securing a private space yielded dissatisfaction with remote assessments. Some caregivers expressed difficulty with regard to the child exhibiting abnormal behavior in the presence of other family members owing to the lack of privacy during remote assessment.

Subsequently, the factors correlated with overall satisfaction were examined using the Spearman rank sum test. In children, the results indicated that factors associated with satisfaction included “seamless hearing” (*r*=0.315; *P*=.09), “visual clarity” (*r*=0.501; *P*<.001), “seamless expression of oneself” (*r*=−0.357; *P*=.03), “feel warmth and human touch” (*r*=−0.254; *P*=.04), “reduced burden of hospital visits” (*r*=0.357; *P*=.03), “ability to act naturally” (*r*=0.514; *P*<.001), and “conners scores” (*r*=−0.292; *P*=.02) reflecting lower ADHD severity. The Mann-Whitney *U* test showed that girls had higher satisfaction levels than boys (*P*=.04), and children with a primary diagnosis of ADHD reported higher satisfaction (*P*=.005).

Factors correlating with overall satisfaction among caregivers were examined using the Spearman rank sum test. The results showed that the factors associated with caregivers’ satisfaction included “seamless hearing” (*r*=0.308; *P*=.01), “visual clarity” (*r*=0.503; *P*<.001), “ability to express oneself” (*r*=−0.341; *P*=.004), and “the child’s ability to act naturally and as usual” (*r*=0.284; *P*=.02). No correlations were found between categorical factors.

Finally, a multiple regression analysis including these factors showed that, for children, “visual clarity” (β=.361; *t*_53_=3.353; *P*=.001), “reduced burden of hospital visits” (β=.238; *t*_53_=2.475; *P*=.02), and “ability to speak naturally and as usual” (β=.243; *t*_53_=2.272; *P*=.03) were crucial factors that influenced their level of satisfaction (adjusted *R*²=0.508). For caregivers, “visual clarity” (β=.352; *t*_63_=2.954; *P*=.004) and “the child’s ability to act naturally and as usual” (β=.223; *t*_63_=2.086; *P*=.04) were found to be significant (adjusted *R*²=0.265).

## Discussion

### Principal Findings

This study evaluated the satisfaction levels with telepsychiatry among children with ADHD and ASD and their caregivers and identified factors associated with satisfaction. Satisfaction with telepsychiatry was high, with 88% (60/68) of caregivers reporting being “satisfied” or “very satisfied.” The acceptance of telehealth remained favorable despite the lower satisfaction rate (47/67, 70%) among children. For children, the factors most strongly associated with high satisfaction included “seamless visibility,” “reduced burden of travel,” and “ability to speak naturally and be themselves.” For caregivers, the items most closely related to high satisfaction were “seamless visibility” and “the child being able to behave naturally and as they usually would.”

The children and caregivers reported high satisfaction with the “seamless hearing” and “clarity of the video,” confirming the technical reliability of telepsychiatry tools. These findings are consistent with those of previous studies [15], suggesting that the technical aspects of telepsychiatry significantly contribute to overall satisfaction.

In previous studies, symptom severity was associated with satisfaction. This study did not yield similar results. However, the study results showed that children with weaker ADHD traits reported higher levels of satisfaction. This result contradicts that of a previous study, suggesting that the children in this study were in stable condition during treatment. When ADHD symptoms were less severe, the children found it easier to concentrate, even in a web-based setting with various environmental distractions at home, which might otherwise hinder engagement.

The responses also indicated that the children experienced significant relief and satisfaction from the reduced time burden associated with travel. This is in agreement with that of a previous study [16]. Considering the long-term nature of treatment for children with neurodevelopmental disorders, reducing caregiver burden is crucial.

Responses from the free text indicated that caregivers had to consider the time for the patient’s appointment and the travel time while managing other children’s schedules, highlighting the difficulties they experienced. Some caregivers also expressed psychological stress from having to take their children out of school for appointments, reflecting the challenge they faced in balancing medical visits. This was often on weekdays with academic responsibilities. However, with telepsychiatry, appointments can be scheduled after school, eliminating the need to choose between education and medical care, thus reducing the burden on caregivers.

A critical factor that influenced the satisfaction of children and caregivers was their ability to “act naturally” during telepsychiatric assessments. Children who reported feeling less natural or perceived a lack of warmth during remote assessments had lower satisfaction levels. This highlights a potential limitation of telepsychiatry, where the absence of direct human interaction can negatively impact the patient experience. Previous studies highlighted similar concerns, underscoring the importance of addressing the psychological aspects of telehealth to improve patient engagement [5]. Additionally, it has been established that using telehealth as a supplementary tool after a relationship has been established with an assessor or physician is essential.

The assessment of this “natural behavior” was based on subjective self-report or caregiver report using a Likert scale, inherently limiting the objective understanding of specific behavioral manifestations in a remote setting. Therefore, incorporating objective behavioral observation (eg, clinician ratings or video analysis) in future research is important to better define this critical concept and inform the development of more effective remote assessment practices.

As represented in the free-text questionnaire, caregivers faced difficulty sustaining their psychological safety during the assessment interviews when discussing sensitive topics, owing to the fear of being overheard by children in the adjacent room. This is a crucial consideration in many Asian countries, particularly in small households and high household density. When securing a sufficiently soundproof private room at home is impossible, it is crucial to provide an external setting that allows the child and caregiver to be separated and conduct the assessment in a safe and secure environment.

### Limitations

This study has several limitations. First, the sample size was insignificant, and the data were collected from specific hospitals in Japan, limiting the generalizability of the results. Second, the results were based on the extent to which telepsychiatric assessments were conducted only once. The satisfaction with using telepsychiatry over a longer period was unclear. Third, the participants in this study were children who had already been diagnosed, and as stated in the inclusion criteria, were relatively stable with no plans to start new medications or psychotherapy within the next 3 months. Therefore, these findings may not be applicable to cases in which the child is undiagnosed and attending consultation for the first time or to patients in the acute phase. Fourth, a selection bias existed because the participants in this study were individuals who were already interested in remote assessments or telepsychiatry. Fifth, while smartphone ownership rates are high in Japan, a significant portion of the population remains unfamiliar with digital devices, as noted by the concept of “digital divide.” Therefore, caution must be exercised when generalizing these results.

### Comparison With Prior Work

Compared with previous studies, Reisinger et al [15] reported that 88% of caregivers of children with ASD were satisfied with remote assessments. Similar to this study, high satisfaction was observable. However, the participants were limited to children with ASD and their caregivers. Galvin et al [16] reported that 62.3% of caregivers of children with ADHD felt comfortable with telemedicine, whereas the children’s comfort level was significantly lower at 34.4%. This study offers novelty compared to previous studies by directly evaluating the satisfaction of children themselves.

In this study, factors associated with children’s satisfaction included “seamless viewing of the screen,” “reduction in the burden of clinic visits,” and “the ability to speak naturally.” For caregivers, “seamless viewing of the screen” and “the child’s ability to behave naturally” influenced their satisfaction. This finding aligns with those of Reisinger et al [15], which also showed that satisfaction is affected by technical reliability and seamless use.

However, children with mild ADHD symptoms were reportedly more satisfied. This point was not indicated in previous studies, suggesting that telemedicine is challenging for children with difficulty concentrating.

This study focused on Japan and demonstrated the acceptability of telemedicine in contexts with different cultural and social backgrounds. Considering many previous studies focused on Western countries, data from Japan are valuable and may contribute to improving health care access and reducing the burden on caregivers. Moreover, this study demonstrated that children with mild ADHD symptoms were more satisfied with telemedicine, a finding that has not been reported in previous studies.

### Conclusions

This study examined the acceptance of remote evaluations by end users, particularly children with ASD or ADHD and their caregivers, identifying the factors associated with higher satisfaction. Each child and their caregiver underwent two assessment sessions, one face-to-face and the other via telepsychiatry (a remote video tool), in a randomized order. The results revealed that telepsychiatry is an effective and practical option for children with ADHD and ASD and their caregivers. Technical reliability and reduced travel burden are the crucial factors that contribute to satisfaction. Applying the findings of this study to clinical practice requires strengthening the support system to help children feel at ease and experience warmth during remote assessments, which is essential for enhancing the quality of telepsychiatric services, particularly for children with pronounced ADHD symptoms. With suitable environmental adjustments, telepsychiatry can be a valuable supplementary option.

## Supplementary material

10.2196/69791 Checklist 1 CONSORT checklist.

## Abbreviations

ADHD: attention-deficit/hyperactivity disorder
ASD: autism spectrum disorder
CONSORT: Consolidated Standards of Reporting Trials

## References

[1] Aoki A, Niimura M, Kato T (2021). Trajectories of healthcare utilization among children and adolescents with autism spectrum disorder and/or attention-deficit/hyperactivity disorder in Japan. Front Psychiatry.

[2] Zahid S, Bodicherla KP, Eskander N, Patel RS (2020). Attention-deficit/hyperactivity disorder and suicidal risk in major depression: analysis of 141,530 adolescent hospitalizations. Cureus.

[3] Cummings JR, Lynch FL, Rust KC (2016). Health services utilization among children with and without autism spectrum disorders. J Autism Dev Disord.

[4] Kuhlthau K, Payakachat N, Delahaye J (2014). Quality of life for parents of children with autism spectrum disorders. Res Autism Spectr Disord.

[5] Baweja R, Soutullo CA, Waxmonsky JG (2021). Review of barriers and interventions to promote treatment engagement for pediatric attention deficit hyperactivity disorder care. World J Psychiatry.

[6] Penner M, Anagnostou E, Ungar WJ (2018). Practice patterns and determinants of wait time for autism spectrum disorder diagnosis in Canada. Mol Autism.

[7] McKenzie K, Forsyth K, O’Hare A (2015). Factors influencing waiting times for diagnosis of Autism spectrum disorder in children and adults. Res Dev Disabil.

[8] Rosen V, Blank E, Lampert E (2022). Brief report: telehealth satisfaction among caregivers of pediatric and adult psychology and psychiatry patients with intellectual and developmental disability in the wake of COVID-19. J Autism Dev Disord.

[9] Bailey RK, Owens DL (2005). Overcoming challenges in the diagnosis and treatment of attention-deficit/hyperactivity disorder in African Americans. J Natl Med Assoc.

[10] Liao X, Lei X, Li Y (2019). Stigma among parents of children with autism: a literature review. Asian J Psychiatr.

[11] Weiss JA, Tint A, Paquette-Smith M, Lunsky Y (2016). Perceived self-efficacy in parents of adolescents and adults with autism spectrum disorder. Autism.

[12] Gabellone A, Marzulli L, Matera E (2022). Expectations and concerns about the use of telemedicine for autism spectrum disorder: a cross-sectional survey of parents and healthcare professionals. J Clin Med.

[13] Shaker AA, Austin SF, Storebø OJ (2023). Psychiatric treatment conducted via telemedicine versus in-person modality in posttraumatic stress disorder, mood disorders, and anxiety disorders: systematic review and meta-analysis. JMIR Ment Health.

[14] Hagi K, Kurokawa S, Takamiya A (2023). Telepsychiatry versus face-to-face treatment: systematic review and meta-analysis of randomised controlled trials. Br J Psychiatry.

[15] Reisinger DL, Hines E, Raches C, Tang Q, James C, Keehn RM (2022). Provider and caregiver satisfaction with telehealth evaluation of autism spectrum disorder in young children during the COVID-19 pandemic. J Autism Dev Disord.

[16] Galvin E, Gavin B, Kilbride K (2024). The use of telehealth in attention-deficit/hyperactivity disorder: a survey of parents and caregivers. Eur Child Adolesc Psychiatry.

[17] Kurokawa S, Nomura K, Hosogane N (2024). Reliability of telepsychiatry assessments using the attention-deficit/hyperactivity disorder rating scale-IV for children with neurodevelopmental disorders and their caregivers: randomized feasibility study. J Med Internet Res.

